# Medical and Philosophical Intelligence

**Published:** 1813-10

**Authors:** 


### MEDICAL AND PHILOSOPHICAL INTELLIGENCE.

*Description of a Resinous Substance lately dug out of the Earth at Highgate.*—By THOMAS THOMSON, M.D. F.R.S.

DURING the last attempt to make a tunnel through Highgate-hill, in the neighbourhood of London, a very considerable number of curious fossils were discovered. The beds dug through consisted partly of gravel and partly of clay. The number of shells thrown out, and the round masses of lime-stone, could not escape the most careless observer; but one of the most remarkable substances detected was a resinous body, in shapeless masses of various sizes.

The colour of Highgate resin is of a dirty yellowish light brown. It is semitransparent. Its lustre is resinous, and its surface smooth; though not perfectly so; but having the appearance of having been rubbed, as would have happened had it been mixed with gravel upon the margin of the sea-shore, or a lake.—Brittle; not so easily broken as common resin; but much more so than copal: softer than copal; has a resinous and aromatic smell, especially when heated; this smell is peculiar, though it has some faint resemblance to the smell of camphor.

When heated it melts, and may be rendered as liquid as water without alteration in its colour. It catches fire at the flame of a candle, and burns with a clear yellow flame, and emitting abundance of smoke, as is the case with other resins. At the same time it emits a strong aromatic colour.

When in lumps it is insoluble in all the reagents I tried, namely, water, alcohol, potash ley, acetic acid; except ether, nitric acid, and sulphuric acid, which act upon it more or less.

Ether renders it opake, and white, and quite tender; so that it has lost its cohesion, and crumbles into powder upon the least pressure between the fingers. The ether at the same time dissolves a portion of it which it deposites, and becomes milky when agitated with water.

Nitric acid acts upon it slowly when assisted by heat, and partly dissolves it, and partly converts it into a red colored substance. The acid itself becomes red, and when diluted with water lets the resin again fall in white flocks. These flocks when dry are in the state of a light yellow-colored powder, having a bitter taste. I could not dissolve it in water; but it dissolved in alcohol, at least as easily as the unaltered resin.

Sulphuric acid readily chars this resinous body when assisted by heat.

When reduced to the state of a fine powder, alcohol readily dissolves a small portion of it, and lets it fall again when mixed with water; but alcohol is a bad solvent of this resinous body. The same observations apply to ether.

I cannot find that either potash or subcarbonate of potash dissolve this resin, though boiled with it for some time in the state of powder. This is the property which distinguishes Highgate resin from every other with which I am acquainted. Even amber is partially acted upon by alkaline leys, and tinges them yellow very speedily.

Nor do I find that acetic acid dissolves any perceptible portion of this resin after a week's digestion in it, when in the state of a fine powder. I even triturated them together for a considerable time in a mortar, and then boiled them in a glass tube, but no solution was effected.

I have not tried the action of oils upon Highgate resin; but from the properties above described, I conceive there is reason to presume that, like copal, it will not dissolve in any of them.

It burns all away before the blow-pipe upon a piece of metal without leaving any perceptible ash behind it, when we make choice of pieces quite free from any earthy matter attached to them.

Such are the properties of this substance, as far as I have examined them. They are sufficient, I think, to distinguish it from all the vegetable substances hitherto observed. It approaches nearest to copal and amber; but is distinguished from the first by its solution in alcohol, and its non-solution in potash ley; from the second, by its readily melting when heated, and by its melting without any perceptible change of its properties. Thus the chemical properties of this singular substance throw no light upon the source from which it was derived; and cannot, therefore, facilitate our inquiries into the revolutions which the southern part of this kingdom has undergone, and the various animal and vegetable remains so thickly scattered in its bowels.

---

*Antimonial Acids.*—No metal has been subjected to a more persevering examination than antimony. But its chemical properties are so difficult of investigation, that the most accurate and ingenious chemists have contradicted one another in their most recent experiments. Thenard found six oxides of antimony, Proust reduced them to two, and Berzelius makes them four. Theory is certainly in favour of the last opinion. The two oxides of antimony containing most oxygen possess, according to Berzelius, the properties of acids. He calls them *antimonious* and *antimonic acids*. The antimonious acid is white. The *calx antimonii elota* of the old pharmacopœias is this acid combined with potash, and containing an excess of acid. When boiled in water a neutral antimonite of potash is obtained, from which other antimonites may be formed. The antimonite of barytes crystallizes in white needles, and is not altered by exposure to the air.

The antimonic acid has a straw yellow colour. It is formed by heating antimony in nitro-muriatic acid. Berzelius has examined several of the saline combinations which it forms with the different bases.

The two acids of antimony have the property of combining together, and of uniting likewise with the other oxides of that metal. This makes it so difficult to examine the oxides of antimony with accuracy. (Berzelius, *Lärbok i Kemien*, ii. 159.)

---

*Acid of Tin.*—The peroxide of tin has been repeatedly considered as an acid by chemists. Bergman and Guyton Morveau have each published dissertations on the subject. Berzelius has lately examined the matter anew; and though he admits that it possesses some characters in common with acids, yet he thinks that they are not sufficient to entitle it to the name.

*Acid of Tellurium.*—The oxide of tellurium, according to the observations servations of Berzelius possesses at once the characters of an acid and a base. Hence it is capable of combining with bases and forming salts, while at the same time it unites with acids, and forms another kind of saline compound. On that account it may be either called *telluric acid* or *oxide of tellurium.*

---

*Sulphite of Copper.*—There are two oxides of copper, the red and the black; the first composed of 1 atom of metal and 1 atom of oxygen, the second of 1 atom of metal and 2 atoms of oxygen. The red oxide refuses to combine with sulphuric acid. When the two substances are brought in contact, the red oxide of copper divides itself into two equal portions, one of which gives all its oyxgen to the other; so that one half of the oxide is reduced to the metallic state, and one half converted into black oxide. This last half unites with the sulphuric acid, and forms common sulphate of copper. The black oxide refuses to combine with sulphurous acid. When the two substances come in contact, the black oxide gives out half its oxygen, and by this means is changed into red oxide, while a portion of the sulphurous acid is changed into sulphuric acid.

Sulphite of copper is of a red colour, and crystallizes. It may be obtained by mixing sulphite of potash and sulphate of copper together, or by passing a current of sulphurous acid gas through water, in which black oxide of copper is suspended. It is decomposed by heat and by boiling in water. According to the experiments of Chevreul, it is composed of

| | |
|---|---|
| Red oxide of copper . . . . . . . . . | 63·84 |
| Sulphurous acid . . . . . . . . . . . | 36·16 |
| | 100 |

Dr. Thomson observes, that, this analysis cannot be accurate. It gives us, he says, 100 acid + 176·549 oxide. Now the weight of an integrant particle of sulphurous acid is 4, and of red oxide of copper 9. Hence if the two substances unite particle to particle, the compound must consist of 100 acid + 225 oxide. We learn from the experiments of Berzelius that sulphuric and sulphurous acids unite with the same weight of base. Now, according to him, 100 sulphuric acid unite with 183 of red oxide of copper. (*Ann. de Chim.* lxxvii. 83.) Hence 80 sulphurous acid would unite with 183 red oxide. This gives us 100 acid + 228·75 red oxide, which agrees very nearly with an atom of acid and an atom of oxide. Hence Dr. T. conceives it to be indisputably much nearer the truth than Chevreul's analysis. For Chevreul's experiments see Ann. de Chim. vol. lxxxiii. p. 181.

Chevreul obtained likewise a triple salt, composed of sulphurous acid, potash, and red oxide of copper. He procured it by mixing a cold solution of sulphite of potash with nitrate of copper. The triple salt precipitated of a yellow color. According to his analysis, it is composed of

| | |
|---|---|
| Red oxide . . . . . . . . . . . . | 0·9360 |
| Potash . . . . . . . . . . . . . . | 0·1556 |
| Acid . . . . . . . . . . . . . . . | 0·6270 |
| | 1·7186 |

 *A simple*

*A simple mode of obtaining a very intense heat.*—Dr. Marcet, of Guy's Hospital, has lately discovered an easy and convenient method of producing, on a small scale, a degree of heat which has hardly ever been exceeded. The process consists in urging the flame of a lamp of spirit of wine by a current of oxygen gas. The apparatus required for the purpose consists of a tin vessel, or gas holder, from which a small jet of oxygen gas is forced out, with some degree of violence, by the introduction of water through a funnel one or two feet in length. If a diamond be exposed to the flame of a lamp, thus acted upon by the jet of gas, it burns, and disappears in a few minutes. Platina wire of moderate thickness, is instantly melted; and globules of this metal, weighing as much as four or five grains, can thus be obtained in quick succession. During this process of fusion, a scintillation of the metal is observed, as if it was undergoing combustion; but this appears to be owing to minute particles of melted platina, which are simply dispersed by the intensity of the heat. Iron wire is burnt by this means with a degree of rapidity and brilliancy which even exceeds that of Ingenhouz's striking mode of burning iron wire in oxygen gas; and small needles of quartz are readily melted and vitrified by the same means.

---

Officers of the Massachusett's Medical Society, elected at the annual meeting in June, 1812.

John Warren, M.D. *President.*
Joshua Fisher, M.D. *Vice-President.*
David Townsend, A.M.
Thomas Welsh, M.D.
Aaron Dexter, M.D. } *Censors.*
Josiah Bartlett, M.D.
William Spooner, M.D.
Thomas Welsh, M.D. *Corresponding Secretary.*
John C. Warren, M.D. *Recording Secretary.*
John Fleet, M.D. *Librarian.*
John G. Cotten, M.D. *Treasurer.*

Hon. Oliver Fiske, Drs. Jonathan Osgood, Thomas Babbit, Abraham Haskell, Austin Flint, *Censors of the District Society in Worcester.*

---

The Medical Society of London resumed its sittings for the season on Monday the 27th instant.

---

A medical practitioner of great respectability, and extensive practice in a central part of London, has requested, through our medium, to make known his wish to form a partnership, with a view of entirely relinquishing business at the expiration of a period thought sufficient for establishing his successor. A premium adequate to the advantages will be required, and to prevent unnecessary trouble, no gentleman need apply unless he can command 1800*l.* in the first instance.

Particulars may be known by applying to Mr. Royston, Princes'-street, Cavendish-square, either personally, or by letters post paid.

Dr.

Dr. Barton, whose Elements of Botany have been most favorably received in America, is preparing for the press, a Flora of the State of Virginia; a Prodromus of a Flora of the States of New York, New Jersey, Pennsylvania, Delaware, Maryland, and Virginia; a work on the Geography of North American Trees and Shrubs; an elementary work on Zoology; a volume on the Indians of North America; and on the third part of Collections towards a Materia Medica of the United States. Some of these works are speedily to appear!

---

Mr. Singer has in the press, "Elements of Electricity and Electro-Chemistry, being a synopsis of the existing state of electrical knowledge. It will appear at the commencement of the ensuing year.

---

Mr. W. Henley, Member of the London Philosophical Society, is preparing for the press a series of Chemical Tables, intended to exhibit the properties of all the present known bodies, the result of their union, the composition of the oxides, acids, and their compounds, with the effects produced by the action of heat, light, and electricity; the whole forming a complete abstract of the science of chemistry.

---

The following gentlemen have obtained the degree of Doctor in Medicine from the University of Glasgow within the last twelve months:—Mr. Rowland Lawrence, from England.—Mr. William George Burrell, from Scotland.—Mr. James Tennant, from Scotland.—Mr. George Forsyth, from Ireland.—Mr. William M'Keowan, from Ireland.—Mr. Alexander Brown, from Scotland.—Mr. William Penman, from Scotland.—Mr. James Kennedy, from Scotland.—Mr. Thomas Coulson Carpenter, from England.—Mr. William Walkinshaw, from Scotland.—Mr. Thomas Stoddart, from Scotland.

---

*Medical School of St. Thomas's and Guy's Hospitals.*—The Winter Course of Lectures at these adjoining hospitals, will commence the beginning of October, viz.

*At St. Thomas's.*—Anatomy and the Operations of Surgery, by Mr. Astley Cooper and Mr. Henry Cline.—Principles and Practice of Surgery, by Mr. Astley Cooper.

*At Guy's.*—Practice of Medicine, by Dr. Babington and Dr. Curry. —Chemistry, by Dr. Babington, Dr. Marcet, and Mr. Allen.—Experimental Philosophy, by Mr. Allen.—Theory of Medicine, and Materia Medica, by Dr. Curry and Dr. Cholmeley.—Midwifery, and Diseases of Women and Children, by Dr. Haighton.—Physiology, or Laws of the Animal Œconomy, by Dr. Haighton.—Structure and Diseases of the Teeth, by Mr. Fox.

N.B. These several Lectures are so arranged, that no two of them interfere in the hours of attendance; and the whole is calculated to form a complete course of medical and chirurgical instruction. Terms and other particulars may be learnt at the respective hospitals.

---

*Anatomical Theatre, London Hospital.*—On Friday, October 1, at twelve o'clock, Dr. Dennison, F.A.S. member of the Royal College of Physicians, London, will recommence his Lectures on the Theory and Practice of Midwifery, and the Diseases of Women and Children.

---

*Anatomical Theatre, Bristol.*—Mr. Thomas Shute will commence his Winter Course of Lectures on Anatomy, Physiology, and the Principles of, and Operations in, Surgery, on Friday, October 1, at eight o'clock in the morning.

---

*University of Glasgow.*—The Medical Lectures in the University of Glasgow, will begin on Monday, the 1st of November, at the following hours:—Institutions of Medicine, by Dr. Freer, at half past eight in the morning.—Surgery, by Dr. Jeffray, at ten.—Midwifery, by Mr. Towers, at eleven.—Practice of Medicine, by Dr. Freer, at twelve.—Anatomy, by Dr. Jeffray, at two o'clock in the afternoon.—Dietetics, Materia Medica, and Pharmacy, by Dr. Millar, at three.—Chemistry, and Chemical Pharmacy, by Dr. Cleghorn, at seven.

Clinical Lectures on the cases of patients in the Royal Infirmary, the first Lecture on Thursday, the 11th of November, at six o'clock.

Dr. Brown will commence his Lectures on Botany, about the beginning of May next.

---

Mr. T. J. Pettigrew, F.L.S. Member of the Royal College of Surgeons of London, will commence a Course of popular Lectures on Human Anatomy and Physiology, on Wednesday the 20th of October, at eight o'clock in the evening precisely, and they will be continued every Wednesday and Friday at the same hour.

---

*Theatre of Anatomy, Windmill-street.*—Plan of a Course of Lectures on Chemical Philosophy, by William Thomas Brande, F.R.S. Prof. Chem. R. I.—These lectures commence on the second Tuesday in October, at nine in the morning, and are continued every Tuesday, Thursday, and Saturday throughout the season, terminating in May.

The subjects comprehended in the course are treated of in the following order:

Division I.—*Of the Powers and Properties of Matter, and the general Laws of Chemical Changes.*

§ 1. Attraction, crystallization, chemical affinity, laws of combination and decomposition.

§ 2. Light and heat, their influence as chemical agents in art and nature.

§ 3. Electricity, its laws and connection with chemical phenomena.

Division II.—*Of Undecompounded Substances, and their mutual Combinations.*

§ 1. Substances that support combustion, oxygene, chlorine.

§ 2. Inflammable and acidifiable substances, hydrogene, nitrogene, sulphur, phosphorus, carbon, boron.

§ 3. Metals, and their combinations with the various substances described in the earlier part of the course.

Division

Division III.—*Vegetable Chemistry.*

§ 1. Chemical physiology of vegetables.
§ 2. Modes of analysis, ultimate and proximate elements.
§ 3. Processes of fermentation, and their products.

Division IV.—*Chemistry of the Animal Kingdom.*

§ 1. General views connected with this department of the science.
§ 2. Composition and properties of the solids and fluids of animals, products of disease.
§ 3. Animal functions.

Division V.—*Geology.*

§ 1. Primitive and secondary rocks, structure and situation of veins.
§ 2. Decay of rocks, production of soils, their analysis, and principles of agricultural improvement.
§ 3. Mineral waters, methods of ascertaining their contents by tests and by analysis.
§ 4. Volcanic rocks, phenomena and products of volcanic eruptions.

In the first division of the course, the principles and objects of chemical science, and the general laws of chemical changes, are explained; and the phenomena of attraction, and of light, heat, and electricity developed, and illustrated by numerous experiments.

In the second division, the undecompounded bodies are examined, and the modes of procuring them in a pure form, and of ascertaining their chemical characters, exhibited upon an extended scale. The lectures on the metals include a succinct account of mineralogy, and of the methods of analysing and assaying ores.

This part of the course will also contain a full examination of pharmaceutical chemistry; the chemical processes of the pharmacopœiæ will be particularly described, and compared with those adopted by the manufacturer.

The third and fourth divisions of the course relate to organic substances. The chemical changes induced by vegetation are here inquired into; the principles of vegetables, the theory of fermentation, and the characters of its products, are then examined.

The chemical history of animals is the next object of inquiry: it is illustrated by an examination of their component parts, in health and in disease; by an inquiry into the chemistry of the animal functions, and into the application of chemical principles to the treatment of diseases.

The course concludes with an account of the structure of the earth, of the changes which it is undergoing, of the objects and uses of geology, and of the principles of agricultural chemistry.

The applications of chemistry to the arts and manufactures, and to economical purposes, are discussed at some length in various parts of the course; and the most important of them are experimentally exhibited.

---

Dr. Ramsbotham will commence his Lectures on the Science and

Practice

Practice of Midwifery, on Monday, the 4th of October, at his house, No. 9, Old Jewry. Pupils will have the advantage of a valuable museum.

---

Dr. Adams's hour of lecturing is ten o'clock, and not twelve, as was printed in our last.

---

Medical and Chemical Lectures, St. George's Hospital, and George-street, Hanover-square, will commence on Monday, October 4th, at the usual morning hours, viz. the Lectures on the Materia Medica and Practice of Physic at eight, and the Chemical at a quarter after nine o'clock. By George Pearson, M.D. V.R.S. Senior Physician of St. George's Hospital, of the College of Physicians, &c. —A register is kept of the cases of Dr. Pearson, in St. George's Hospital, and an account given of them every Saturday morning at nine.

---

Dr. Squire will, on Tuesday the 5th of this month, begin a Course of Lectures on the Theory and Practice of Midwifery, and the Diseases of Women and Children.

---

Dr. Hooper and Dr. Ager will begin their next Course on the Theory and Practice of Physic, Chemistry, and the Materia Medica, at the Theatre, 26, Cork-street, Burlington Gardens, on Monday, October 4th, 1813, at eight o'clock in the morning.

Three Courses are given every year, beginning in February, June, and October, each on the first Monday of the month.

---

Mr. A. Carlisle, F.R.S. F.A.S. Professor of Anatomy in the Royal Academy, and Surgeon to the Westminster Hospital, will begin his Course of Lectures on the Art and Practice of Surgery, and the Sciences connected therewith, on Tuesday, October 12th, at twelve o'clock, at his house in Soho-square.

The introductory discourse is open to all professional students, and the subject to be continued on Tuesdays and Thursdays at the same hour.

The diseases and accidents allotted to the province of surgery will be amply treated of, and illustrated by cases from the lecturer's experience. A compendious view of the animal economy will be adduced to illustrate the several processes of disease and of recovery.

The operations of surgery, and the anatomy of the affected parts, are to be demonstrated.

---

*Theatre of Anatomy, Windmill-street.*—Mr. Brodie will begin his Lectures on the Theory and Practice of Surgery, on Monday, October 4th, at seven in the evening.

---

*St. George's Hospital.*—Sir Everard Home will begin his Course of twelve Lectures on the principal Operations of Surgery (given gratuitously to the pupils of this Hospital) on Thursday the 14th of October.

METEO-

